# Supplementary figures and images for: The structural coverage of the human proteome before and after AlphaFold
Source: PLoS Comput Biol. 2022 Jan 24;18(1):e1009818. doi: 10.1371/journal.pcbi.1009818 (PMC8812986; doi:10.1371/journal.pcbi.1009818)

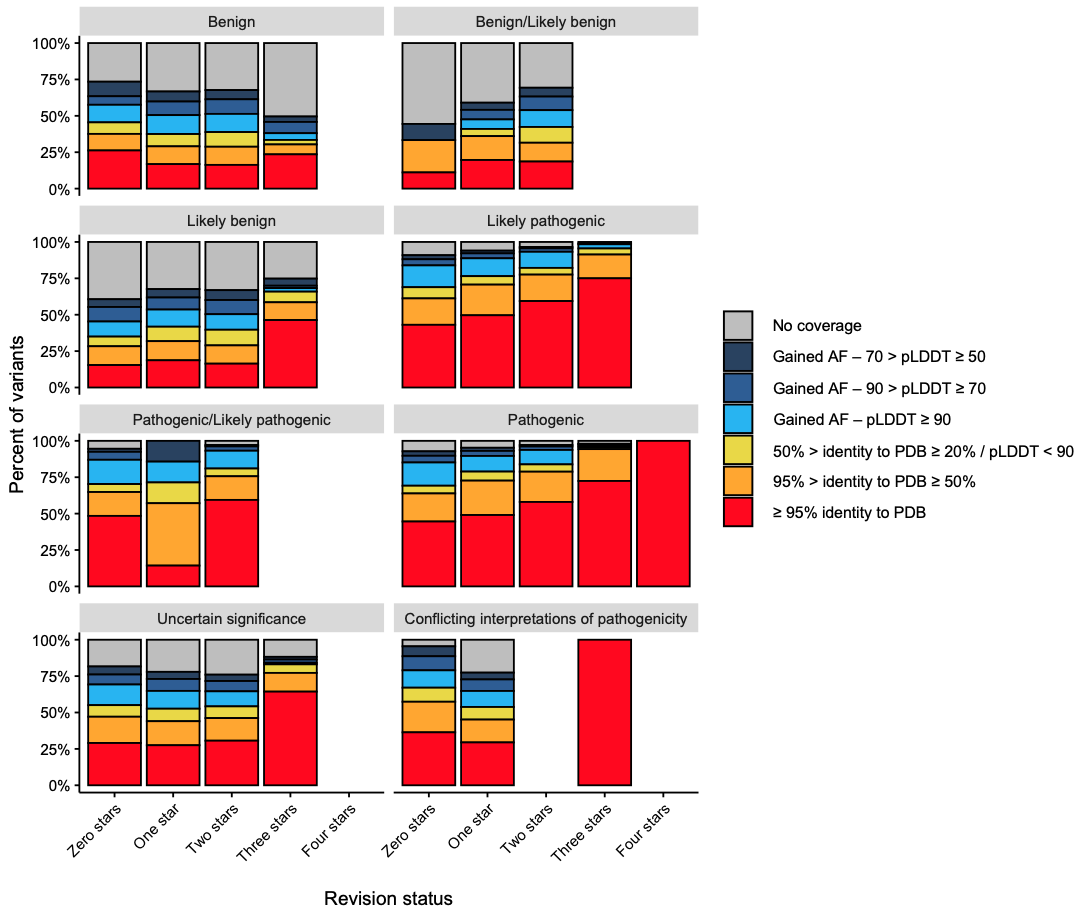

Supplement: S1 Fig — Structural coverage of Clinvar mtuations (y-axis) depending on their pathogenicity (different panels) and their review status (x-axis). (TIFF) [file pcbi.1009818.s001.tiff]

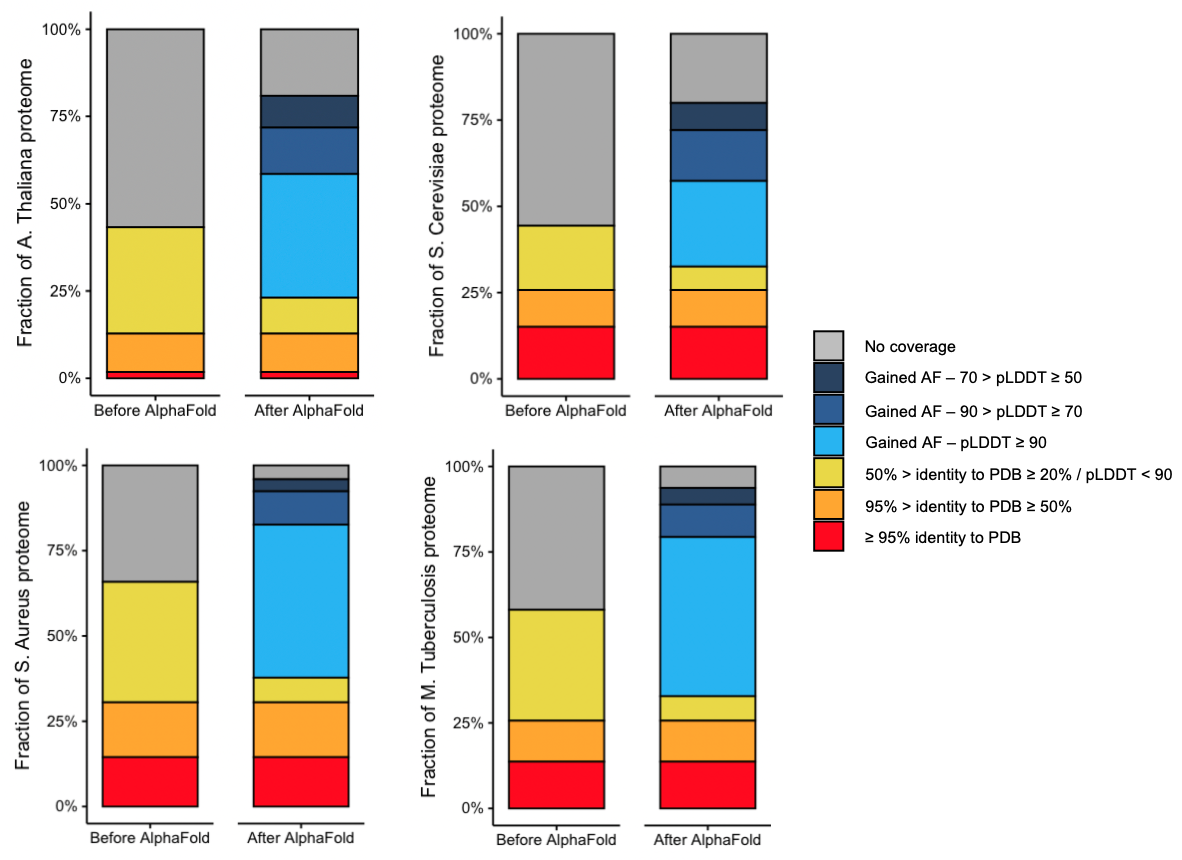

Supplement: S2 Fig — Structural coverage (y-axis) of the proteome of the four different organisms before (left) and after (right) including the AlphaFold models. (TIFF) [file pcbi.1009818.s002.tiff]
