# Supplementary material for: The structural coverage of the human proteome before and after AlphaFold
Source: PLoS Comput Biol. 2022 Jan 24;18(1):e1009818. doi: 10.1371/journal.pcbi.1009818 (PMC8812986; doi:10.1371/journal.pcbi.1009818)
Supplement: Attachment — Submitted filename: Revision_letter.docx [file pcbi.1009818.s003.docx]

Eduard Porta Pardo

Cancer Immunogenomics Laboratory

Barcelona Supercomputing Center (BSC)

Josep Carreras Leukaemia Research Institute (IJC)

Barcelona, Spain

Dear Drs. Tossatto and Elofsson;

We are submitting a revised version of our manuscript “***The structural coverage of the human proteome before and after Alphafold***”. We would like to begin by thanking both the reviewers and editors for their careful feedback and suggestions. We were pleased to see that, in general, the reviewers appreciated our manuscript, while highlighting some points where it could improve.

We believe that this revised version has improved over the previous one, as we have addressed all of the reviewer’s comments. While we provide an explanation below with the details and changes to the manuscript that we have done to address each point, we would like to highlight here how we addressed what we believe were the two major concerns: 1) the impact of AlphaFold models in the structural coverage of non-human organisms and 2) the relationship of this manuscript with the preprint “A structural biology community assessment of AlphaFold2 applications”, in which we are co-authors.

Regarding the first, following the suggestion of Reviewer 2 we have now included in our manuscript the changes in structural coverage for an additional four organisms: *Arabidopsis Thaliana*, *Saccharomyces Cerevisiae, Staphylococcus Aureus* and *Mycobacterium Tuberculosis*. The details of these analyses can now be found in Figure 4 and Figure S2. We chose these four organisms to achieve two goals. First, we wanted to cover both, eukaryotes (*A. Thaliana* and *S. Cerevisiae*) and prokaryotes (*S. Aureus* and *M. Tuberculosis*) to see if we could find any differences. Secondly, we wanted to include clinically relevant bacteria (*S. Aureus* and *M. Tuberculosis*). The reason is that we believe that structural models from AlphaFold could significantly contribute to the identification of new antibiotics, a looming biomedical crisis, and we wanted to quantify the impact that they could have.

Regarding the relationship of our manuscript with the community preprint, our contributions to both manuscripts are intrinsically different. In the community preprint we provided the comparison between RoseTTAfold and AlphaFold models for PFAM domains, something that is not covered in this manuscript except for a sentence in the discussion where we claim that this is something that we believed should be done (at the time of the initial submission the community preprint was in the very early first stages). We have now, in fact, updated this sentence to explain our results in the other manuscript.

Moreover, while in the community preprint there are some general comparisons between Swissmodel and AlphaFold, the community preprint does not go into the detail that we provide in this manuscript for the human proteome. Moreover, it does not give any detailed analyses or comparisons for different protein families like we do for disease-associated, essential or cancer driver proteins. Finally, the community preprint does not provide any insights about the coverage for disease-associated mutations.

All of the above already prove, in our opinion, that both scientific contributions are substantially different and merit independent publications. That being said, and as explained above, we now also include a section about the contribution of AlphaFold to the coverage of other organisms, a subject not addressed at all in the community preprint, further proving that this manuscript merits its own publication.

Overall, we would like to thank again the Reviewers for their comments. We honestly think that the paper has improved by adding the comparison with other species along with the rest of comments. We hope that we have successfully addressed the Reviewer’s concerns and that the manuscript is now acceptable for publication.

Yours sincerely;

Eduard Porta Pardo, on behalf of the authors

The comments by the reviewers were generally positive, with a few (mainly technical) issues remaining to be fixed. It has been argued that the paper would benefit from using more organisms for a comparative assessment of the distribution of classes. One reviewer also notes that the code used to generate the data is not available.

It has been noted that, while the paper was under review, the authors have contributed to a preprint which covers roughly the same topic.

See: <https://www.biorxiv.org/content/10.1101/2021.09.26.461876v1>

In light of this, the authors should argue why they believe this submission to be different from their contribution to that paper. Expanding the scope of the paper, e.g. by adding more organisms, would be an obvious answer. The final call on this will have to be made by the Editorial Office according to journal policy.

We appreciate the comments from both, the reviewers and editors. As explained in the cover letter, there are substantial differences between the community preprint and this manuscript. The community preprint focuses more on applications of AlphaFold in general. This manuscript, on the other hand, focused only in the impact in human proteins and mutations, allowing us to explore this topic in much more detail. That being said, we now include a section comparing the results in human and four other species: *A. thaliana, S. cerevisiae, S. aureus* and *M. tuberculosis*

Reviewer's Responses to Questions

**Comments to the Authors:**

**Please note here if the review is uploaded as an attachment.**

***Reviewer #1:*** Porta-Pardo et al. in the article “The structural coverage of the human proteome before and after AlphaFold” provided an analysis aiming at describing the state-of-the-art about our current capacity of inferring the structure of the human proteome. They compared and measured experimentally-derived structures, template-based homology models and artificial intelligence predictions as provided recently by the AlphaFold2 method and available in AlphaFoldDB.

The manuscript is of interest as it can provide a good reference for all discussions about the available knowledge of human protein structures. The language is simple and clear and the methodology used is fair. However, there are a couple of issues that need to be clarified or modified and that are critical for the presented statistics and in my opinion prevent acceptance of the manuscript in the current form.

Major

My main concern is about the definition of disordered residues which is provided by the IUPred-long tool. That choice is never justified nor is it discussed that the tool tends to underpredict disorder. The authors mentioned CASP results to justify the use of AlphaFold over other prediction tools, however they didn’t do the same for disorder predictors. They could cite the recent Critical Assessment of Intrinsic protein Disorder (CAID) and mention that IUPred is a good compromise between speed and accuracy even though it isn’t in the top ten (PMID: 33875885).

In terms of coverage of the human proteome, a statistic is already available from the following MobiDB page, <https://mobidb.org/statistics?proteome=UP000005640>. The page provides a comparison with other disorder prediction tools and takes the same proteome reference used by AlphaFoldDB (Proteome ID UP000005640) which includes all expressed human proteins.

As the reviewer pointed out, we chose IUPred because we share the opinion that it is a good compromise between speed and accuracy. We have now made this point evident in the methods section and cite the appropriate reference.

*New text in the Methods section:*

*We used IUPRed2 because, based on recent benchmarkings, it was a good compromise between speed and accuracy*[*[50]*](https://paperpile.com/c/sBkiwH/3U6v)*.*

The second point is about the definition of the human proteome dataset. The choice of using ENSEMBL proteins and selecting one isoform for each gene is fine but somehow it complicates the reasoning and reproducibility. UniProt offers a great service in managing and cross-referencing ENSEMBL and other databases. Under the human proteome page (<https://www.uniprot.org/proteomes/UP000005640>) it is possible to retrieve all human proteins which are expressed (78,120 proteins, UniProt version 2021_03). Also, at the same page UniProt provides a link to download representative proteins, one protein per gene (20,600 proteins). The number of representative proteins is very close to the one used by the authors. I suggest using the UniProt definition of the proteome (20,600 representative proteins) in order to be consistent with AlphaFoldDB (<https://alphafold.ebi.ac.uk/download>). I’m pretty sure that it would not change the overall results.

We used ENSEMBL as reference because all the databases that we have about disease-associated mutations (ClinVar, BoostDM, TCGA) are mapped to ENSEMBL protein isoforms. However, we have now updated the proteome, attempting to identify as many matching protein sequences between ENSEMBL and UniProt as possible, which has allowed us to reach 19.250 proteins. We have updated all the analyses with this new reference proteome and, as the reviewer hypothesized, the overall numbers have not significantly changed.

We do not provide the detailed text here as this affects many small parts of the entire paper.

Minor

In the abstract it is not clear whether AlphaFold promotes to structure a fraction of disordered residues or the actual “dark residues”, or both. In other words it is not clear why the structured residues (75%) does not sum up with the 22% of the dark proteome.

We thank the reviewer for raising this point which was not clear. Note that the numbers that we are going to say below reflect the updated percentages, as we now have expanded the number of proteins up to 19,250.

AlphaFold models do contribute to both, IDRs and dark proteome, as evidenced in Figure 1f. When we only consider the PDB data in our analysis, the percentage of residues without structure coverage, PFAM domains or predicted to be disordered, i.e.: the dark proteome, is 26%. If we include the models from AF2, this number is reduced to 10%, because AF contributes with structural models for more than half of the dark proteome (Figure 1f).

We have introduced a small modification in the abstract to make this point more explicit as we consider it is important to highlight not only the fact that the protein structural coverage is now higher thanks to AF2, but also that the structural uncertainty is reduced.

*New text in the Abstract:*

*At the same time the fraction of “dark proteome” is reduced from 26% to just 10% when AlphaFold models are considered.*

Figure 2. I suggest explaining why the 3 example structures have been selected. That is well explained in the manuscript but not in the caption. Also showing what are the residues which were not covered by any structure before AlphaFold would help to understand why the method was useful for those cases. In the end the pLDDT is not that useful for those examples as they are almost completely predicted with a very high score.

The caption of Figure 2 has been modified and now it indicates that the 3 selected examples do not have a structure in PDB nor any template. About the second part of the comment, we believe that this point is addressed as all the selected proteins do not have structure coverage at all in any of the residues before the AF2 models.

*New text in the caption of Figure 2*

***e-g)*** *AlphaFold models for previously structureless AGMO, DEGS1 and PEMT proteins.*

***Reviewer #2:*** The paper by Eduard Porta-Pardo etal is a conceptually straightforward but still well executed exploration of how AlphaFold has changed our ability to have good structural models in the human proteome. It adds to the work done by the AlphaFold team and importantly provides a third party view of this, with a focus on disease causing genes as well as overall statistics.

There is nothing major which causes an issue or concern here (ie, the science is solid), however, I think this sort of exploration would be far more powerful is extended to other organisms with AlphaFold predictions, as I think the contrast between the human scenario (with more experimental focus) and other organisms would be important. As I suspect the big issue will be on just importance/impact of this paper, I think this will make for a more rounded and impactful paper.

We now include a section about changes in coverage in four other organisms: *Arabidopsis thaliana*, *Saccharomyces cerevisiae*, *Staphylococcus aureus* and *Mycobacterium tuberculosis*. The details of the analysis can be found in that section, in Figure 4, Figure S2 and in the Discussion. In summary, the impact of AlphaFold models is significantly higher in non-human organisms. In particular, in the case of bacteria, the high-quality structural data added by AlphaFold covers around 60% of the proteome, compared to only ~35% for humans.

We do not provide the detailed text here as it is an entire new results section of the paper and a paragraph in the discussion. Both are highlighted in red in the revised manuscript, though.

Minor issues:

I think the authors could have introduced their >95%, 50-95% and 20-50% identity buckets better in the text; One has to draw lines arbitrarily somewhere, but they should be up front about this in the text as well as in the figures. This is more stylistic than anything else.

We thank the reviewer for highlighting this aspect. We have added two sentences in the first paragraph of the first section of the results explaining the choice of the 3 sequence identity ranges. We also specified these identity buckets in the caption of Figure 1.

*New text in the Results section “***The structural coverage of the human proteome before AlphaFold”**

*Additionally, for a more straightforward interpretation of the results, we set in this study 3 different levels of protein structural coverage according to 3 ranges of sequence identity percentages between a protein and a PDB chain. The ranges are: >95%, which includes the real structure of the protein; 95-50%, which contains useful templates to study the impact of mutations*[*[25]*](https://paperpile.com/c/sBkiwH/BnKj)*; and 50-20%, which contemplates structural coverage of distant related proteins assuming that structure is more conserved than sequence.*

Another point, the authors use the European convention of . for separator with thousands, whereas I think plos expects the US/UK convention of comma.

The text has been modified accordingly and now includes the US/UK convention of comma.

***Reviewer #3:*** AlphaFold is a deep learning algorithm developed by Google's DeepMind that predicts protein structure from its amino acid sequence. The AlphaFold database containing the predictions for the human proteome and 20 other organisms has now been released to the community. This paper evaluates the impact of the AlphaFold database on our knowledge of human proteins. It also evaluates the impact on the knowledge of disease-associated genes and mutations. Lastly, the authors provide examples of some disease-associated proteins where AlphaFold provided key insights. They conclude that the AlphaFold has largely closed the gap between the sequence-structure knowledge gap.

Strengths:

i) Extensive evaluation of the coverage of human proteome before and after AlphaFold. Databases used - PDB, ENSEMBL

* The amount of protein for which no structure information was available has reduced from 53% before AllphaFold (Fig. 1d) to 25% after AlphaFold (Fig. 1e)

* AlphaFold reduced the number of proteins without structural coverage from 4,832 to only 29

* GO enrichment analysis on these 4,832 proteins revealed strong enrichment in 269 olfactory receptor genes, 66 lipid metabolism genes, 1700 genes integral to membrane, and 1056 genes without previous information.

ii) Extensive evaluation of the improvement of our knowledge of the disease related genes and mutations extracted from - DisGeNet, OncoKB, TCGA, ClinVar

* The coverage of disease-related genes based on their PDB structures (50-61%) is higher than that of the average proteome (47%). Thereforefore, it is not surprising that AlphaFold does not contribute as much to them as it does to the rest of the proteome

* AlphaFold increased the percentage of high quality coverage proteins from 31% to 43% for benign mutations of ClinVar, and from 71% to 83% for pathogenic mutations

ii) Good examples of the disease-causing genes B3GALT6, MED12, in which AlphaFold has revealed the specific-clustering due to disease-causing mutations

The authors conclude that the after AlphaFold has "practically completed" our coverage of the human foldable proteome, and this shall open the door for more challenging problems in structural biology.

Weakness:

i) Parts of the paper of were very hard to follow and need relatively extensive re-writing and tightening

We have tried to improve the writing in some of the sections that we considered more difficult to follow.

ii) The mutations taken from ClinVar were reported as benign or pathogenic. The authors need to provide criteria they used to select those variants. Normally, variants with at least one star would be selected. Otherwise, the results could be polluted by noise.

The reviewer raises a very interesting point. In our analysis we used all the Clinvar missense variants, without filtering by review criteria. We now provide the details of the results when splitting the variants by review criteria in Figure S1. The results show the same pattern: benign mutations have lower PDB coverage than pathogenic mutations across all review stages. As a matter of fact, there seems to be a positive correlation between the PDB coverage of pathogenic variants and their review status. For example, all pathogenic variants with four stars have an experimental PDB structure, whereas only 50% of the pathogenic variants with zero stars do.

*New text in the Results section*

*Notably, the review status of Clinvar mutations does not affect the conclusions of our analysis (****Figure S1****). In fact, there seems to be a positive correlation between the PDB coverage of pathogenic variants and their review status: all pathogenic variants with four stars have an experimental PDB structure, whereas only 50% of the pathogenic variants with zero stars do.*
